# Association between out-patient visits and air pollution in Chiang Mai, Thailand: Lessons from a unique situation involving a large data set showing high seasonal levels of air pollution

**DOI:** 10.1371/journal.pone.0272995

**Published:** 2022-08-18

**Authors:** Tunyathron Varapongpisan, Till D. Frank, Lily Ingsrisawang

**Affiliations:** 1 Department of Statistics, Faculty of Science, Ramkhamhaeng University, Bangkapi, Bangkok, Thailand; 2 Department of Psychology and Department of Physics, University of Connecticut, Storrs, Connecticut, United States of America; 3 Department of Statistics, Faculty of Science, Kasetsart University, Chatuchak, Bangkok, Thailand; James Cook University, AUSTRALIA

## Abstract

Chiang Mai is one of the most known cities of Northern Thailand, representative for various cities in the East and South-East Asian region exhibiting seasonal smog crises. While a few studies have attempted to address smog crises effects on human health in that geographic region, research in this regard is still in its infancy. We exploited a unique situation based on two factors: large pollutant concentration variations due to the Chiang Mai smog crises and a relatively large sample of out-patient visits. About 216,000 out-patient visits in the area of Chiang Mai during the period of 2011 to 2014 for upper (J30-J39) and lower (J44) respiratory tract diseases were evaluated with respect to associations with particulate matter (PM_10_), ozone (O_3_), and nitrogen dioxide (NO_2_) concentrations using single-pollutant and multiple-pollutants Poisson regression models. All three pollutants were found to be associated with visits due to upper respiratory tract diseases (with relative risks RR = 1.023 at cumulative lag 05, 95% CI: 1.021–1.025, per 10 μg/m^3^ PM_10_ increase, RR = 1.123 at lag 05, 95% CI: 1.118–1.129, per 10 ppb O_3_ increase, and RR = 1.110 at lag 05, 95% CI: 1.102–1.119, per 10 ppb NO_2_ increase). Likewise, all three pollutants were found to be associated with visits due to lower respiratory tract diseases (with RR = 1.016 at lag 06, 95% CI: 1.015–1.017, per 10 μg/m^3^ PM_10_ increase, RR = 1.073 at lag 06, 95% CI: 1.070–1.076, per 10 ppb O_3_ increase, and RR = 1.046 at lag 06, 95% CI: 1.040–1.051, per 10 ppb NO_2_ increase). Multi-pollutants modeling analysis identified O_3_ as a relatively independent risk factor and PM_10_-NO_2_ pollutants models as promising two-pollutants models. Overall, these results demonstrate the adverse effects of all three air pollutants on respiratory morbidity and call for air pollution reduction and control.

## Introduction

Air pollution is a major threat to human health, in general, and to the human respiratory system, in particular. Various studies have examined the association between air pollution levels and mortality and morbidity [1–6]. Of particular interest has been the relationship between respiratory diseases and particulate matter concentrations. Particulate matter concentration in the air have been associated with respiratory tract diseases in several studies of the East and South-East Asian region reporting from China, South Korea, and Taiwan [7–9]. These associations have been found for PM_10_ [8] as well as for PM_2.5_ [7, 9]. They have been found in studies based on short observation periods of just on year [7, 9] or longer periods that cover more than 10 years [8]. They have been found for upper and lower respiratory tract diseases [7–9]. In summary, the association between particulate matter levels and respiratory diseases is a robust phenomenon.

Several cities in the East and South-East Asian region are known to suffer from severe, seasonal violations of air quality standards such as Seoul in Korea [10], Beijing, Shanghai, Guangzhou, Wuhan, Xi’an in China [11–14], Tiachung in Taiwan [15], Johor Bahru and Pasir Gudang in Malaysia [16, 17]. Chiang Mai, one of the largest cities in the northern part of Thailand is one of those cities. Since 2007, Chiang Mai suffers from an annual smog crisis around the month of March [18–22]. In the past, in March, PM_2.5_ particulate matter concentrations have reached daily peak values that were 5 times higher than the 25 μg/m^3^ value recommended by the WHO [19] and PM_10_ daily concentration values have climbed up to values 4 times higher than the WHO recommended daily PM_10_ value of 50 μg/m^3^ [21]. Recently, in March 2019, the US AQI determined for Chiang Mai climbed up to a record level of 300 such that Chiang Mai became the world’s leading city on the top 10 list of cities with the worst air pollution [23].

The smog crisis in Chiang Mai has fueled interest in systematic research targeting the air pollution in Chiang Mai and consequences for human health [24]. While this kind of research is important in its own merit, Chiang Mai should also been seen as a testbed for studying implications of severe, seasonal air pollution episodes on the human health as observed in various cities in the East and South-East Asian region (see above). Regardless of the motivation, research evaluating the Chiang Mai smog crisis is still in its infancy and has produced conflicting results.

The city district of Chiang Mai and the surrounding districts have been the focus of various studies [19, 20, 25, 26]. While all studies reported adverse effects of air pollution on human health, they also reported conflicting results. Specifically, adverse effects of PM_10_ have been reported for pulmonary morbidity by Ruchiraset and Tantrakarnapa [20] and Pothirat et al. [26]. However, they were not found in the study by Wiwatanadate [25] on upper respiratory tract diseases. Moreover, conflicting results as far as the role of NO_2_ is concerned were reported. Ruchiraset and Tantrakarnapa [20] examined pneumonia hospitalizations in the city district of Chiang Mai that had been reported during a 12 years period from 2003 to 2014. They showed that not only PM_10_ but also NO_2_ were positively associated with pneumonia cases. Wiwatanadate [25] studied a sample of about 3000 participants, who were living in a suburban district of Chiang Mai, for a 4 months period. Again, it was found that various symptoms of upper respiratory tract diseases were positively associated with NO_2_ concentrations. In the study by Pothirat et al. [26] hospitalization visits due to acute exacerbation of chronic obstructive pulmonary disease (AECOPD) of the J44.1 category were analyzed in a rural district to the north of Chiang Mai during 2016 and 2017. In contrast to the studies by Ruchiraset and Tantrakarnapa [20] and Wiwatanadate [25] no association between AECOPD visits and NO_2_ concentrations was found. Pongpiachan and Paowa [19] conducted an analysis on a relative long period from 2007 to 2013. Patient hospitalizations in the city district of Chiang Mai were investigated with respect to possible associations with NO_x_. In contrast to all three aforementioned studies, they found that increases in NO_x_ concentration decreased (rather than increased) morbidity.

Finally, the studies by Ruchiraset and Tantrakarnapa [20] and Pothirat et al. [26] did not support the adverse effects of O_3_ on the respiratory system that have been documented in various other studies around the world (see, e.g., [1, 4, 27, 28]). Even more strikingly, the studies by Wiwatanadate [25] and Pongpiachan and Paowa [19] reported that O_3_ levels decrease respiratory tract disease morbidity which is counter-intuitive as it was to some extent acknowledge by Wiwatanadate [25]. In summary, although there is an increasing interest in studying the high-risk situation in Chiang Mai, the role that the air pollutants PM_10_, NO_2_, and O_3_ play in this situation is still unclear.

Thus, the goal of the present study is to focus on the Chiang Mai smog problem and estimate the dependency of the morbidity to diseases of the upper and lower respiratory tracts on PM_10_, NO_2_, and O_3_ air pollutants with the help of Poisson regression models, on the one hand, and a relatively large data set of daily data observed over several years, on the other hand. To this end, diseases of two subcategories of respiratory tract diseases were considered for which patient data was made available by the Ministry of Public Health of Thailand. In order to work with a relatively large data set, patient data from the whole region around Chiang Mai that included the Chiang Mai city district was evaluated. A specific objective of our study was to determine the time pattern of associated risks.

## Material and methods

### Hospital out-patient data

We used out-patient visits from Chiang Mai as indicator of morbidity in Chiang Mai. More precisely, daily visits of walk-in patients (i.e., patients who came from outside and were released on the same day) to public hospitals of the Chiang Mai province were considered. Those hospitals were under the management of the Ministry of Public Health (MOPH) of Thailand. Data were obtained from the Strategy and Planning Division [29] of the Office of the Permanent Secretary of the MOPH. The received data file was anonymized. The data covered a three years period in 2011–2014 starting with October 2011. This period was selected because it falls in the period after the beginning of the smog crisis in 2007 (see Introduction) and it comes with data that had been collected under a new health system in October 2011 that was able to account for a larger portion of actual out-patient visits as compared to the pre-2011 data collection system. Out-patients visits were classified by the MOPH according to the International Classification of Diseases, 10th Revision (ICD-10; World Health Organization, Geneva). Visits related to two subcategories of respiratory disease of the upper and lower respiratory tract were evaluated: the category J30-J39 for other diseases of the upper respiratory tract and the category J44 for other chronic obstructive pulmonary diseases.

### Environmental data

Daily measurement data of air pollution and weather variables were used as environmental data. Pollutant concentrations were used from the years 2011–2014 as collected by the Pollution Control Department of the Ministry of Natural Resources and Environment of Thailand [30]. In this study we focused on the pollutants PM_10_ (as measured in μg/m^3^), O_3_ (as measured in ppb), and NO_2_ (as measured again in ppb). Air pollution data from two detectors located in the Chiang Mai city district was used. On the scale of the entire Chiang Mai province, the detectors were located relatively close to each other. Therefore, the data from the two detectors was averaged. The approximate location of the two detectors (i.e., the location of the Chiang Mai city district). The Chiang Mai city district is a densely populated metropolitan area. Daily meteorological data such as temperature (in Celsius), pressure (in hPa), and humidity (as relative humidity in %) for the same period were retrieved from the Thai Meteorological Department of the Ministry of Digital Economy and Society of Thailand [31]. The data collection site was at the Chiang Mai city district again. The TMD took for temperature, pressure and relative humidity eight measurements (in 3 hours intervals) each day and determined out of those values daily maximum scores that were used in our study.

### Modeling approach

A Poisson regression model [32, 33] was used to determine associations between daily counts of out-patient visits in the J30-J39 and J44 categories and the three aforementioned air pollutants PM_10_, NO_2_, and O_3_. SO_2_ concentrations as used in other studies (e.g., Deng et al. [34]) have not been used because explorative analysis showed that they have been relatively low (see S1 Table 1 in S1 File). In line with previous studies from the South-Asian region [27, 28, 35, 36] and from Chiang Mai in particular [19, 20, 26, 37, 38] meteorological variables such as temperature and relative humidity were added to the model to control for possible confounding effects. Pressure was also included (as in Pothirat et al. [26]) because explorative correlation analysis showed a strong correlation between pressure and out-patient visits. Explorative analysis revealed also that visits were considerably lower on Saturdays and Sundays as compared to weekdays. Note that in general Thai people tend to visit public hospitals less frequently on weekends because public hospitals run only an emergency schedule on weekends [39]. Moreover, visits counts were considerably lower in the month of July (which was considered to be the central month of the Thai rainy season) as compared to the remaining months of the year. Therefore, the day of the week (weekend day versus workday) and the month of the year (month of July versus remaining months) were taken into account as confounding variables (for a similar approach see e.g. [8]). In order to determine the time pattern of associated risks, delayed exposure effects were taken into account by considering lagged variables [40]. More precisely, single pollutant regression models used lagged pollutant variables (lag 0 to lag 6) and cumulatively lagged pollutant variables (lag 01 to lag 06). In summary, the regression models were defined by

$$log\left[E\left(Y_{t}\right)\right]=\beta_{1}\;Z_{t-s}+\beta_{2}\;DOW+\beta_{3}\;MOY+\sum_{m=1}^{3}\gamma_{m}\;C_{m}+\alpha$$ (1)

In Eq (1) *Y*_*t*_ was the number of visits of the category J30-39 or J44 on day *t*. *Z*_*t-s*_ with coefficient *β*_1_ was the pollutant concentration under consideration on day *t-s*, where *s* denoted the lag with *s* = 0,…,6. DOW and MOY denoted day-of-the week (weekend day versus workday) and month-of-the-year (month of July versus remaining months) variables, respectively, as defined above, with coefficients *β*_2_ and *β*_3_. *C*_*m*_ denoted meteorological variables given by relative humidity (*C*_1_), temperature (*C*_2_), and pressure (*C*_3_) with coefficients γ_1_, γ_2_, and γ_3_. α was the intercept. The cumulatively lagged pollutant models were defined by Eq (1) with *Z*_*t-s*_ replaced by *Z*(cum,*t*,*s*). The latter was defined by *Z*(cum,*t*,*s*) = (*Z*_*t*_+ *Z*_*t-*1_ + … + *Z*_*t-s*_)/(*s* +1) again with *s* = 0,…,6. With the help of *β*_1_, relative risks (RRs) and the corresponding 95% confidence intervals were calculated for a change in morbidity for 10 μg/m^3^ increases in PM_10_ and 10 ppb increases in NO_2_ and O_3_, respectively. Since it has been suggested that health conditions of individuals are frequently the effect of a set of interacting pollutants [14] or the effect of a set of pollutants coming from a particular source [41], by analogy to Eq (1), multi-pollutant models were constructed involving two of the three pollutants or all three pollutants considered in our study.

## Results

### Data description

**Fig 1:**
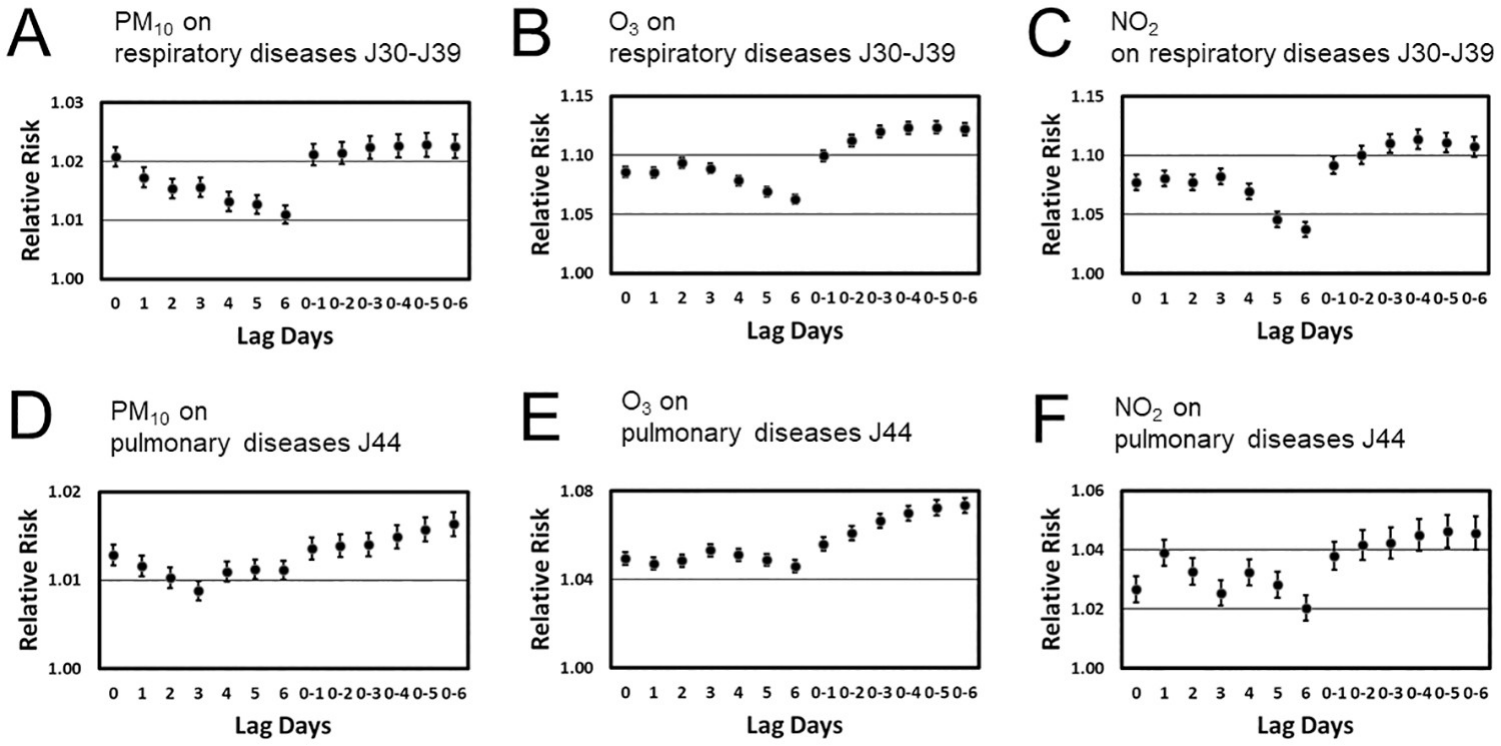
Relative risk estimates obtained from single pollutant models with different lag days. Panels A, B, and C: Effects of PM_10_, O_3_, and NO_2_ on visits due to upper respiratory tract diseases of the J30-J39 category. Panels D, E, and F: Effects of PM_10_, O_3_, and NO_2_ on visits due to pulmonary diseases (i.e., lower respiratory tract diseases) of the J44 category.

In the observation period a total of 67530 visits of the J30-J39 category (with M = 62 visits per day, SD = 53) and a total of 148494 visits of the J44 category (with M = 136 visits per day, SD = 93) were recorded. During that period PM_10_ scores were at M = 74.6 μg/m^3^ (SD = 55.2), O_3_ scores were at M = 24.8 ppb (SD = 14.0), and NO_2_ scores were at M = 49.9 ppb (SD = 24.7), see Table 1. A graph of the PM_10_ concentrations over time can be found in S1 Fig 1 in S1 File. A more detailed statistical characterization of the air pollutants can be found in S1 Table 1 in S1 File. The figure illustrates that PM_10_ concentrations showed seasonal high values during the months February, March and April. During the three seasons covered in the current study, the peak values (observed in March) were about 300, 300, and 400 μg/m^3^, respectively, and clearly violated air pollution standards. The mean temperature during the observation period was 33.0 °C (SD = 2.9), relative humidity assumed a mean level of 88.0% (SD = 6.8), and pressure was at a mean level of 1012 hPa (SD = 4), see Table 1.

**Table 1:** Statistics for health and environmental variables as observed in the Chiang Mai province, Thailand, during October 2011 to September 2014.

Variable	Total or Mean ± SD
*Patients*	
Out-patients visits (total)	216024
J30-J39 related visits (total)	67530
J30-J39 related visits (daily)	62 ± 53
J44 related visits (total)	148494
J44 related visits (daily)	136 ± 93
Age median (range)	60 (0–113)
Male/female visits	51% / 49%
*Air pollutants*	
PM_10_ (daily scores in μg/m^3^)	74.6 ± 55.2
O_3_ (daily scores in ppb)	24.8 ± 14.0
NO_2_ (daily scores in ppb)	49.9 ± 24.7
*Weather conditions*	
Temperature (daily in °C)	33.0 ± 2.9
Relative humidity (daily in %)	88.0 ± 6.8
Pressure (daily in hPa)	1012.0 ± 4.0

Pearson’s correlation coefficients were computed to identify linear relationship between all variables used in our study, see Table 2. All three pollutants were positively correlated with each other. Temperature and pressure were positively correlated with all three pollutants. In contrast, relative humidity showed a negative correlation with all three pollutants. Importantly, the morbidity counts in both categories were positively correlated with each of the three pollutants PM_10_, NO_2_, and O_3_. As far as the confounding meteorological factors were concerned, temperature showed negative correlations with out-patient visits of both categories. However, only the effect on J30-J39 was statistically significant. For pressure and relative humidity positive and negative statistically significant correlations, respectively, with both disease categories were found.

**Table 2:** Pearson’s correlation coefficients between air pollutants, weather variables, and out-patient visits due to upper respiratory tract (J30-J39) and pulmonary (J44) diseases. (*p < .05, **p < .01).

	**PM_10_**	**O_3_**	**NO_2_**	**Temperature**	**Pressure**	**Humidity**	**J30-J39**	**J44**
**PM**_**10**_	1	0.75**	0.76**	0.33**	0.26**	-0.59**	0.26**	0.24**
**O**_**3**_		1	0.71**	0.36**	0.34**	-0.52**	0.29**	0.25**
**NO**_**2**_			1	0.06*	0.45**	-0.31**	0.29**	0.22**
**Temperature**				1	-0.40**	-0.63**	-0.06*	-0.03
**Pressure**					1	0.11**	0.28**	0.24**
**Humidity**						1	-0.07*	-0.07*
**J30-J39**							1	0.80**
**J44**								1

### Regression models

Fig 1 presents the estimated RRs (and 95% CIs) for respiratory (category J30-J39) and pulmonary (category J44) diseases due to 10 μg/m^3^ and 10 ppb increases in PM_10_, O_3_, and NO_2_, respectively, for lags 0 to 6 and cumulative lags 01 to 06. Overall, the RRs described for all three pollutants positive associations with pollutants and out-patient visits of both categories. Moreover, the associations were statistically significant at all lags. For the single day (single lag) models the maximum effect of PM_10_ was observed at lag 0 both for respiratory and pulmonary diseases. O_3_ showed a maximum effect at lag 2 and 3 for respiratory and pulmonary diseases, respectively. The increases in respiratory disease-related visits (J30-J39) due to NO_2_ air pollution showed a plateau for lag 0 to lag 3. For any of those lags the effect was approximately equally strong. In contrast, the effect of NO_2_ on the increase of pulmonary morbidity (J44) was strongest for lag 1. For the respiratory disease category J30-J39 a clear pattern was found for all three pollutants, namely, that RRs decayed monotonically in magnitude at higher lags 4, 5, and 6. The cumulative lag models in general showed stronger effects than the single day (single lag) models. For respiratory J30-J39 disease-related visits plateaus of relatively large RRs were found for all three pollutants at lags 03 to 06. In contrast, for pulmonary J44 disease-related visits RR scores increased more or less monotonically from lags 01 to 06. This was again observed for all three pollutants PM_10_, O_3_, and NO_2_.

Since the cumulative lag 05 was found to be in the aforementioned plateau region from lags 03 to 06 of maximal pollutant effects on visits of the respiratory disease category J30-J39, this lag was used to construct multiple-pollutants models to explain variations in J30-J39 respiratory diseases. Multi-pollutant models for J44 were constructed using the cumulative lagged variables 06.

**Fig 2:**
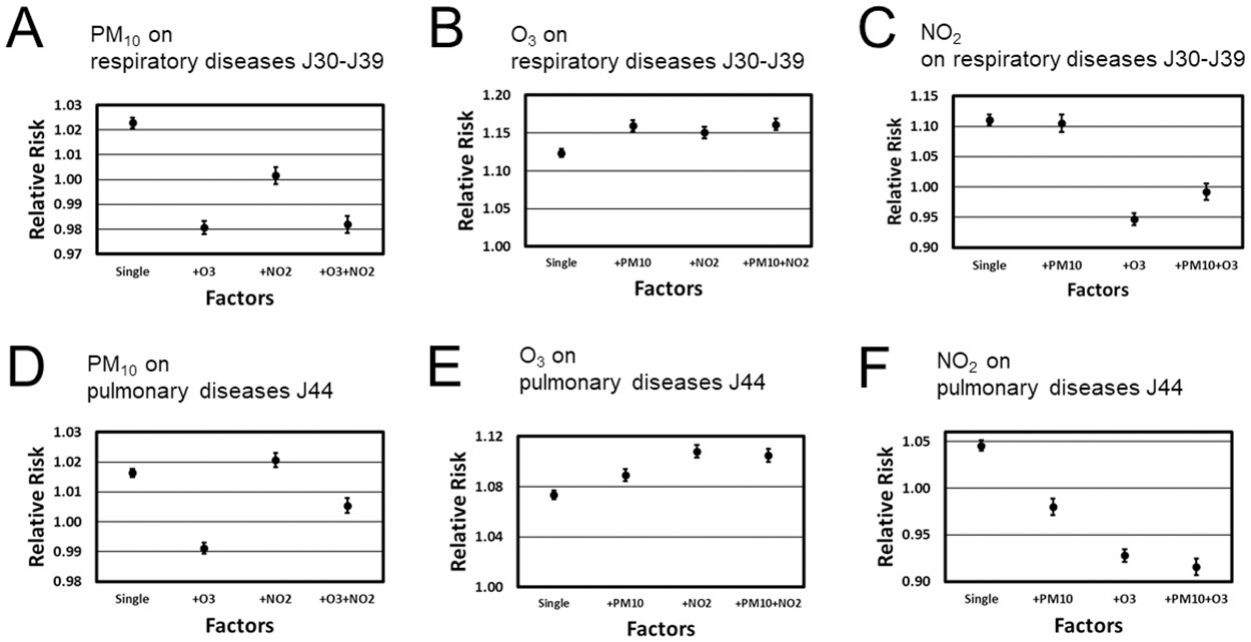
Associations between 10 unit increases in PM_10_, O_3_, NO_2_ concentrations and upper (panels A, B, C) and lower (panels D, E, F) respiratory tract disease cases in the Chiang Mai area as determined by single- and multi-pollutants models as captured in terms of estimated RR factors.

Fig 2 (panels A, B, and C) shows the changes among RRs estimates when comparing single pollutant models with two-pollutants and three-pollutants models. Let us begin with PM_10_ as risk factor for upper respiratory tract diseases (panel A). Due to the influence of O_3_, the positive association between PM_10_ and out-patient visits related to respiratory diseases turned into a negative association. This was observed for the two-pollutants and three-pollutants models involving O_3_ for J30-J39. In contrast, NO_2_ did not affect qualitatively the role of PM_10_ as risk factor. As far as O_3_ was concerned, the role of O_3_ as risk factor with positive association to out-patient visits was not affected by PM_10_ or NO_2_ (panel B). Finally, the impact of NO_2_ on respiratory diseases of the J30-J39 category, as assessed in our study, was affected by O_3_ but not by PM_10_. When taking O_3_ into account, the positive association between NO_2_ and out-patient visits turned into a negative one (panel C).

Panels D, E, and F of Fig 2 show for the (lower respiratory tract) pulmonary disease category J44 similar patterns as observed in panels A, B, and C for the upper respiratory tract disease category J30-J39. The estimated RRs of PM_10_ were qualitatively affected by O_3_ in the two-pollutants model involving PM_10_ and O_3_ such that the positive association was reversed into a negative one. In contrast to the J30-J39 disease category, in the three-pollutants model PM_10_ remained positively associated with pulmonary disease of the J44 category despite the fact that the three-pollutants model involved O_3_ as factor (panel D). O_3_ turned out to be a risk factor for J44 diseases for both the single pollutant and for all constructed multi-pollutants models (panel E). That is, O_3_ was not affected qualitatively by inclusion of the other pollutant factors into the analysis. NO_2_ was only positively associated with J44 diseases for the single pollutant model (panel F). Inclusion of the other pollutants (PM_10_ and O_3_) turned the positive association to a negative one (panel F).

Note that as mentioned above the J30-J39 and J44 multi-pollutants models were constructed using the cumulative lags 05 and 06, respectively. In addition, multi-pollutants models with cumulative lags 04 (for J30-J39) and 05 (for J44) were constructed. Analysis of those models showed qualitative similar results as shown in Fig 2 (see S1 Fig 2 in S1 File).

## Discussion

The association between respiratory diseases and air pollutants was examined for the region of Chiang Mai, which is an exemplary area of areas in the East and South-East Asian region in which populations are exposed to seasonal episodes of unhealthy air conditions (as indicated by air pollutant concentrations that are several times above those values recommended by the WHO).

As shown in Fig 1, the estimated RRs related to PM_10_, O_3_, and NO_2_ concentrations were statistically significant greater than 1 for same day exposure (day 0) and all lagged (day 1 to day 6) and cumulatively lagged (day 01 to 06) effects being considered. Accordingly, being exposed to high air pollutant concentrations on a given day increased the risk of an individual to develop respiratory health problems at the same day, at the next days, or even six days later. As shown in panels A, B, C and D of Fig 1, in general, the risk estimates were smaller at higher lags. More precisely, for upper tract respiratory diseases of the J30-J39 category, RRs related to PM_10_, O_3_, and NO_2_ concentrations at lagged days 5 and 6 were lower as compared to RRs related to same-day pollutant concentrations or PM_10_, O_3_, and NO_2_ concentrations at lagged days 1 to 4 (panels A, B, and C). This might be interpreted to say that the reaction of the respiratory systems as far as diseases of the J30-J39 category are concerned to higher air pollution levels was fairly immediate (reaction at the same day or the following three days). Alternatively, the time patterns presented in panels A, B, C for lag 0 to lag 6 of associated risk indicate that when time elapsed the risk to develop a pollutant-associated disease became lower. The aforementioned time pattern of risks was also observed for diseases of the J44 pulmonary category and PM_10_ (panel D). In contrast, no clear pattern was obtained in this regard for O_3_-associated and NO_2_-associated pulmonary diseases of the J44 category (see panels E and F of Fig 1).

As mentioned in the Results section, RR scores obtained for cumulatively lagged pollutant variables (01 to 06) were in general higher than scores for single day lagged (1 to 6) or same day pollutant variables (compare right and left sides of panels A, B, C, D, E, and F of Fig 1). Accordingly, when air pollutant levels increased by 10 units on average over an extended period (2 days up to 7 days) then health risks for the population of the Chiang Mai area were higher as compared to the situation when air pollutant levels increased by 10 units only on a particular day (lagged day 1 to 6 or same day). According to Fig 2, at least during the observation period from 2011 to 2014, O_3_ acted as an independent risk factor for the population of the Chiang Mai region and the two respiratory disease categories considered in our study in the sense that its role to increase the number of out-patient visits did not change across the single and multiple-pollutants models. Due to this independency, O_3_ may be considered as a particularly useful predictor variable for the occurrence of upper (i.e., category J30-J39) and lower (i.e., category J44) respiratory tract diseases. Furthermore, PM_10_ and NO_2_ (although they were positively correlated, see Table 2) formed a plausible two-pollutants model in which the two pollutants came both with positive associations and predicted together the occurrence of upper respiratory tract diseases of the J30-J39 category in the Chiang Mai population (see Fig 2 panels A and C).

The findings reported above may be used to discuss some conflicting previously reported results from that region and the Chiang Mai area, in particular, as will be shown next.

As indicated in the introduction, in the context of the Chiang Mai smog crises it has previously been reported that high particulate matter concentrations increase the risk for lower respiratory tract diseases such as pneumonia [20] and pulmonary diseases of the J44.1 category [26]. However, while Wiwatanadate [25] examined several symptoms of upper and lower respiratory tracts diseases with respect to possible associations with particulate matter concentrations, support for such associations could not be found. The present study shows clear evidence for PM_10_-associated upper and lower tract respiratory diseases (at least with respect to the J30-J39 and J44 categories). Increased PM_10_ concentrations resulted in an increase of occurrences of respiratory diseases in the Chiang Mai population under consideration. While the aforementioned studies focused on local areas located in the north of Chiang Mai [20, 25] and given by the city district [26], the present study focused on a large scale, namely, the whole Chiang Mai province. Therefore, the results of the present study generalize the previously found findings by Ruchiraset and Tantrakarnapa [20] and Pothirat et al. [26] in the sense that the local phenomena reported by Ruchiraset and Tantrakarnapa [20] and Pothirat et al. [26] are part of a large-scale phenomenon affecting the whole province of Chiang Mai. Moreover, the absence of evidence for PM_10_-associated respiratory health problems in the area studied by Wiwatanadate [25] might be considered as a distinctive characteristics of that area. Finally, as far as adverse effects of PM_10_ concentrations in the air are concerned, in a study by Pothirat et al. [22] mortality due to chronic obstructive pulmonary disease (category J44.9) was found to be associated with PM_10_ concentrations in the Chiang Mai area.–consistent with the results reported in the current study. However, while Pothirat et al. [22] tested single day lagged variables from lag 0 to 7, the association was only statistically significant for lag 6, which means the association was only statistically significant for PM_10_ concentrations 6 days before patients passed away. Although it is difficult to compare pollutant-induced mortality (as in Pothirat et al. [22]) with pollutant-induced morbidity (as in our study), it is interesting to note that our study suggests that there is a whole plateau of equally strong associations at lags 4, 5, and 6 between PM_10_ and visits related to the J44 category, see panel D of Fig 1.

Previous studies devoted to the Chiang Mai smog crisis either reported no association between O_3_ levels and respiratory tract health problems [20, 26] or a negative association in the sense that high O_3_ levels would be beneficial for the human health [19, 25]. The latter is counter-intuitive and contradicts various studies from the East and South-East Asian region [27, 28] and around the globe [1, 4] that suggest that high ozone levels have adverse health effects. In particular, Wiwatanadate [25] acknowledged that such counter-intuitive negative associations might be apparent associations due to the impact of hidden confounding variables. The present study sheds new light on the role of O_3_ levels in the Chiang Mai smog crises. Accordingly, clear evidence has been presented that increased O_3_ levels were associated with increased occurrences of upper and lower respiratory tract diseases in the Chiang Mai population (see panels B and E of Fig 1). Our analysis showed that this effect of O_3_ remains robust even when taking other pollutants such as PM_10_ and NO_2_ into account (see panels B and E of Fig 2). Therefore, the present study not only provides evidence for O_3_-associated respiratory tract diseases related to the Chiang Mai smog crisis but also points out that O_3_ is a useful independent risk factor.

NO_2_ concentrations have been previously examined in the context of the Chiang Mai smog crisis and associations between high NO_2_ levels and various upper respiratory tract health issues [19, 25] and pneumonia (lower respiratory tract) cases [19]. In contrast, Pothirat et al. [26] could not find support for an association between NO_2_ levels and lower respiratory tract problems of the J44.1 pulmonary disease category. The different role of NO_2_ pollutants for lower respiratory tract diseases in the studies by Pongpiachan and Paowa [19] and Pothirat et al. [26] may be explained by acknowledging that the studies considered different disease categories, on the one hand, and focused on different districts. While Pothirat et al. [26] considered a relatively rural district to the north of Chiang Mai, Pongpiachan and Paowa [19] studies the densely populated city district of Chiang Mai. The present study clarifies the role of NO_2_ pollutants for human health in the seasonally air pollution plagued Chiang Mai region. Accordingly, when averaging over or disregarding the impacts of other air pollutants, high NO_2_ concentrations increase the risk for both upper and lower respiratory diseases of the J30-J39 and J44 categories (see panels C and F of Fig 1).

In general, the RR scores for the cumulative lagged variables were higher as compared to the RR scores of the single day variables. This observation may be explained following the arguments by Ding et al. [42]. Accordingly, both biological and behavioral factors may lead to delayed reactions. More precisely, on the one hand, respiratory health problems may need several days to develop and, on the other hand, patients may be reluctant to visit a doctor right away when experiencing symptoms. Rather, patients may delay their decision to seek for medical help. From a modeling perspective, the latter argument is consistent with the assumption that respiratory diseases develop via multi-step processes that involve at least one intermediate (e.g. pre-disease or pre-clinical) state [43]. That is, our data suggest that the two-states schematic presented in the graphical abstract might be considered as a useful minimalistic model but should be refined by more sophisticated models.

The question arises to what extent the results of the present study transfer to other regions within Thailand or the wider East and South-East Asian region. For example, Ostro et al. [44] studied the impact of particulate matter on mortality in Bangkok using data from 1992 to 1995. Mortality due to respiratory diseases was positively associated with PM_10_ concentrations. This type of association was observed for same day PM_10_ concentrations, lagged concentration variables, and cumulatively lagged variables. Likewise, Phosri et al. [45] examined the association between PM_10_ and hospital visits in Bangkok during the 8 years period of 2006 to 2014. Visits related to pneumonia (category J18-J19) and chronic obstructive pulmonary disease (category J40-J47) were found to be associated with increased levels of PM_10_. These findings are similar to those results reported in the present study in panels A and D of Fig 1 with respect to morbidity to respiratory diseases. In this context the question arises whether Bangkok shares with the Chiang Mai region (and other areas of the East and South-East Asian region) the distinctive feature of an annual smog crises. PM_10_ pollutant data observed during the period from 1996 to 2010 reported by Watcharavitoon et al. [46] do not reveal any pronounced annual peaks comparable with the March peaks characteristic for the Chiang Mai smog crises. Having said that the data reported by Watcharavitoon et al. [46] demonstrate the washout effect of the rainy season on PM_10_ concentration as has been observed in the current study for the Chiang Mai region (see Material and methods).

The present study exploits a unique situation that involves, on the one hand, air pollutants that vary over relatively large range of concentrations on a year-to-year basis (related to an annual smog crisis), and, on the other hand, a data set that corresponds to a relatively large sample (about 200,000 patient visits). Such situations have been considered in the health sciences as prime opportunities to estimate relative risks [5]. As mentioned in the introduction, several cities and areas in the East and South-East Asian region suffer from a similar annual smog crisis and, consequently, previous research on this field has evaluated situations involving large-scaled air pollutants variations. However, those studies not necessarily were based on large data sets. For example, Mokoena et al. [14] studied respiratory mortality in the city of Xi’an, China, that features an annual smog crisis [12, 14] similar to the Chiang Mai region. To this end, about 8,000 death cases were considered. We are inclined to say that due to the relative large number of visits considered in our study, the relative variability among the scores was relatively low such that RR estimates obtained in our study exhibited relatively small confidence intervals (see Figs 1 and 2). More precisely, if a RR estimate differed from unity by an amount X, then the confidence interval was typically less than 50% of X. In contrast, in the study by Mokoena et al. [14] confidence intervals were typically multiples of X (up to 10 times the value of X). Based on our results, we speculate that in other studies on regions involving year-to-year air pollution peaks that were based on smaller samples, like the study by Mokoena et al. [14], some RR estimates might have been determined to be not statistically significant due to a lack of statistical power. This line of argumentation may be also supported by the study from Li T et al. [5] on PM_2.5_ associated mortality in Beijing, China. As mentioned in the introduction, Beijing is–just a Chiang Mai—another city in the East and South-East Asian region that suffers from annual episodes of highly polluted air [12]. Li T et al. [5] studied among other things the association between PM_2.5_ and death due to respiratory diseases of the category J00-J99. Specifically, they considered 14,274 death cases of the category J40-J47, 8,141 cases of J09-J18, 3,528 cases of J95-J99, 1,583 cases of J80-J84, and 1,012 cases of J60-J70. Only for the largest sample with 14,274 cases for the J40-J47 category a statistically significant positive association between PM_2.5_ and death rate could be found. For all other (smaller) samples of the remaining disease categories J09-J18, J95-J99, J80-J84, J60-J70 no statistically significant associations could be found. It is striking that all the diseases categories that were tested on the basis of relatively small samples (less than 10,000 cases) produced no statistically significant results, whereas the disease category tested with a relatively large sample (more than 10,000 cases) produced a statistically significant association. Overall, we are inclined to say that these results reported by Li T et al. [5] highlight the need to consider sufficiently large samples, as used in the current study. Finally, as mentioned above, RR scores of cumulative pollutant variables in our study were relatively high, which may indicate that respiratory diseases of the classes considered in our study need some time to develop. Such amplified RR scores of cumulative have also reported by Wang et al. [13] for the population of Shanghai, China. While Wang et al. [13] studied the impact of PM_2.5_ and O_3_ on outpatient visits due to health problems in the upper and lower respiratory tract, our study focused on the PM_10_ and O_3_. Interestingly, Wang et al. [13] could find amplified RR scores for the cumulative PM_2.5_ pollutant variables similar to those reported in our Fig 1 (panels A and D). However, in contrast to our findings (see Fig 1 panels B and E), Wang et al. [13] could not find a significant increase of outpatient visits due to increased O_3_ levels.

Metrological variables were taken into account as confounding variables. To this end, daily extreme values were used. By definition, such daily extreme values are higher than the corresponding daily mean values. Therefore, the question arises to what extend our analysis results depend on the choice of the values for the meteorological values. To answer this question, we conducted the same kind of analysis as reported in the Results section using daily mean meteorological values. We found that the relative risk patterns thus obtained did not differ qualitatively from those reported in the Results section (compare Fig 1 with S1 Fig 3 in S1 File). The only exception was the pattern for the effect of PM_10_ on J30-J39 diseases for single lag model. However, overall, qualitatively, the analysis based on daily meteorological mean values reveals statistically significant effects of all pollutants under consideration on both disease categories. Quantitatively, the relative risk values were lower (compare again Fig 1 with S1 Fig 3 in S1 File). That is, the re-analysis revealed a systematic quantitative (but not qualitative) effect.

Let us briefly address a limitation of the current study. The current study used air pollution data from two detectors located in close proximity to each other that effectively can be considered as a single monitoring site. That is, we did not attempt to capture spatial effects of air pollution concentration. Future studies may try to associate hospitalizations recorded in individual districts of the Chiang Mai province with air pollution data locally measured in those districts. While such a spatial approach can yield insights that go beyond the results obtained in the current study, a particular challenge in this context is to secure data that comes with the appropriate spatial structure.

## Conclusion

The study demonstrated that outpatient visits due to upper and lower respiratory tract diseases of the categories J30-J39 and J44 during the period from 2011 to 2014 were associated with air pollutant levels of PM_10_, O_3_, and NO_2_ in the Chiang Mai area. Importantly, for all tested timing scenarios pollutant concentrations were statistically significant associated with outpatient visits of both disease categories. Therefore, our study supported the conclusion that there was no scenario under which increasing air pollution levels did not have adverse effects on human health and helped to resolve some conflicting results reported earlier. The study pointed out the need for monitoring, publicizing, and controlling air pollutant levels not only in the Chiang Mai area but also in other areas of the East and South-East Asian region characterized by annual smog crisis phenomena similar to the one of Chiang Mai.

## Supporting information

S1 File Supplementary figures and tables. (PDF)
