# Supplementary material for: Association between out-patient visits and air pollution in Chiang Mai, Thailand: Lessons from a unique situation involving a large data set showing high seasonal levels of air pollution
Source: PLoS One. 2022 Aug 18;17(8):e0272995. doi: 10.1371/journal.pone.0272995 (PMC9387779; doi:10.1371/journal.pone.0272995)

S1 Appendix. Supplementary figures and tables

S1 Fig 1 Time series of PM_{10} concentrations during the 3 years observation period.

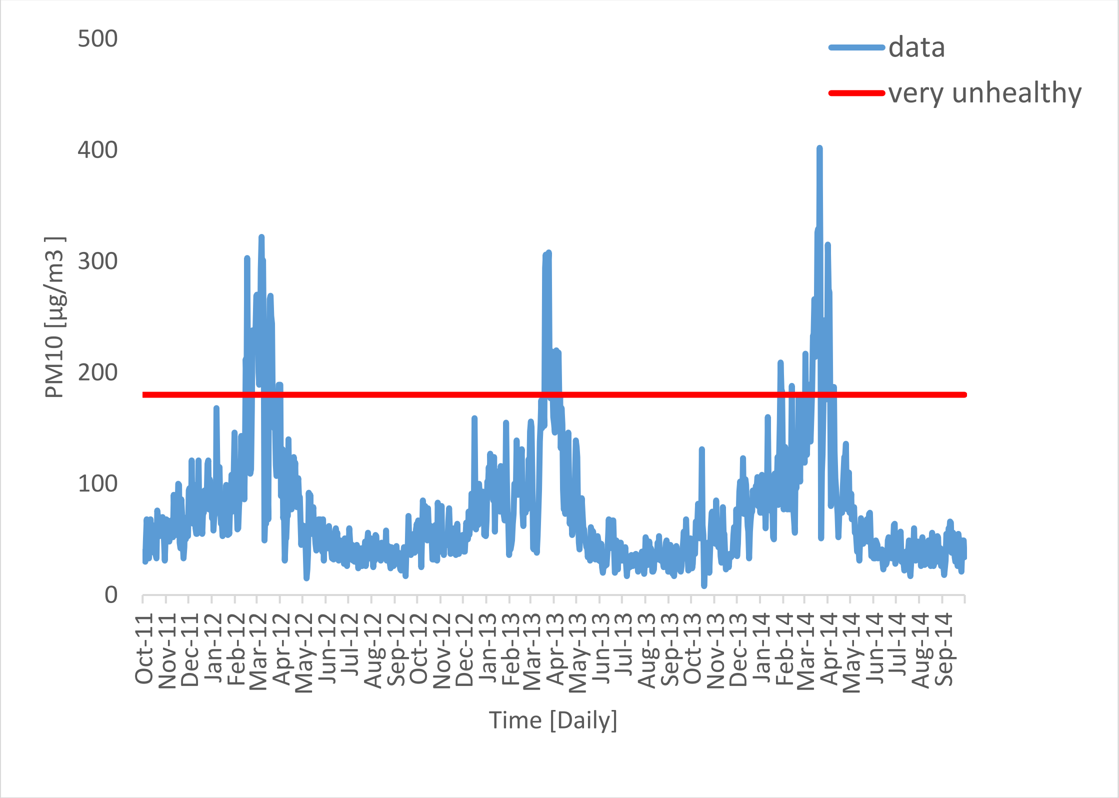

S1 Table 1 Detailed characterization of the pair pollutants PM_{10}, O_3, and NO2. SO_2 is also listed to demonstrate that during the observation period the SO_2 pollutant only ranged in the healthy level.

Variable	Total or Mean ± SD	Minimum	25th percentile	50th percentile	75th percentile	Maximum
Air pollutants						
PM_{10} (daily scores in µg/m^3)	74.6 ± 55.2	8	39	56	90	402
O_3 (daily scores in ppb)	24.8 ± 14.0	5	30	46	67	129
NO_2 (daily scores in ppb)	49.9 ± 24.7	5	15	20	33	82
SO_2 (daily scores in ppb)	1.8 ± 1.2	0	1	1	1	9

S1 Fig 2 Associations between 10 unit increases in PM_{10}, O_3, NO_2 concentrations and upper (panels A, B, C) and lower (panels D, E, F) respiratory tract disease cases in the Chiang Mai area as determined by single- and multi-pollutants models as captured in terms of estimated RR factors. Cumulative lags 04 (for J30-J39) and 05 (for J44) were used.

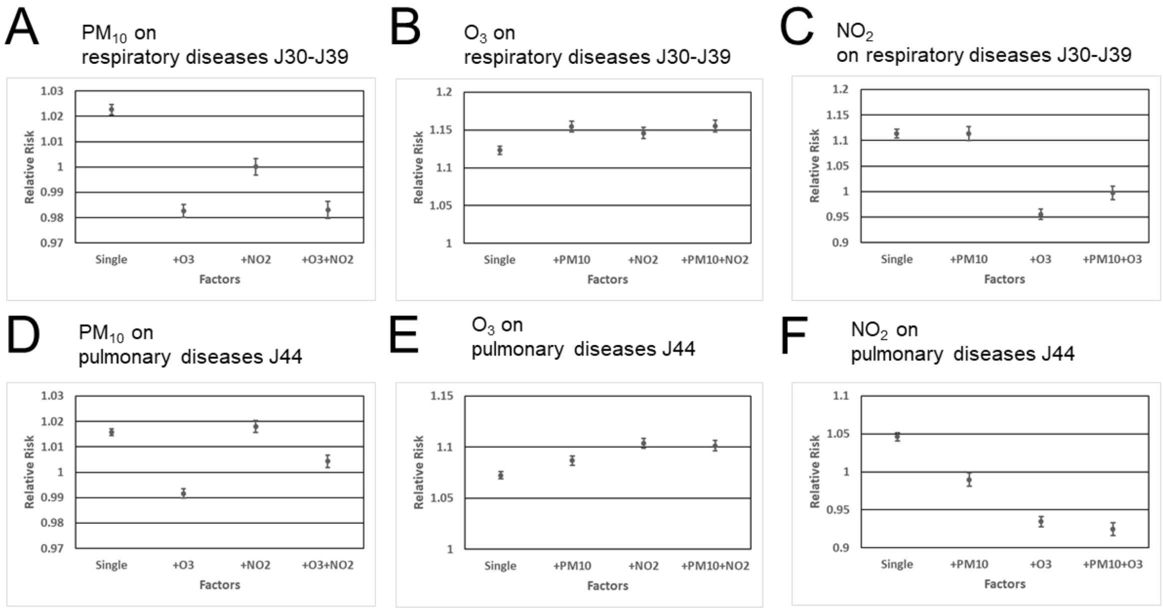

S1 Fig 3 Relative risk estimates obtained from single pollutant models with different lag days. Confounding meteorological variables are taken into account in terms of mean values rather than extreme values. Panels A, B, and C: Effects of PM_{10}, O_3, and NO_2 on visits due to upper respiratory tract diseases of the J30-J39 category. Panels D, E, and F: Effects of PM_{10}, O_3, and NO_2 on visits due to pulmonary diseases (i.e., lower respiratory tract diseases) of the J44 category.

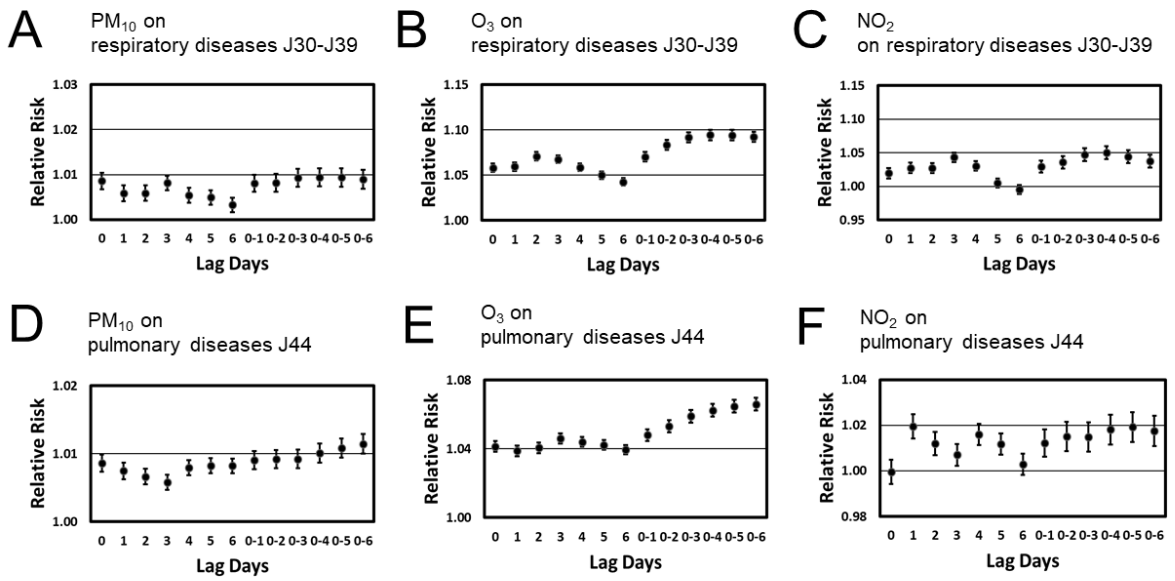

Supplement: S1 File — (PDF) [file pone.0272995.s001.pdf]
